# T-Cadherin (*CDH13*) and Non-Coding RNAs: The Crosstalk Between Health and Disease

**DOI:** 10.3390/ijms26136127

**Published:** 2025-06-26

**Authors:** Kseniya Rubina, Artem Maier, Polina Klimovich, Veronika Sysoeva, Daniil Romashin, Ekaterina Semina, Vsevolod Tkachuk

**Affiliations:** 1 Faculty of Medicine, Lomonosov Moscow State University, 119991 Moscow, Russialex2050@mail.ru (P.K.);; 2 Institute of Experimental Cardiology, National Medical Research Center of Cardiology Named After Academician E.I. Chazov, 121552 Moscow, Russia

**Keywords:** T-cadherin, *CDH13*, non-coding RNA, miRNA, regulation

## Abstract

T-cadherin (*CDH13*) is an atypical, glycosyl-phosphatidylinositol-anchored cadherin with functions ranging from axon guidance and vascular patterning to adipokine signaling and cell-fate specification. Originally identified as a homophilic cue for migrating neural crest cells, projecting axons, and growing blood vessels, it later emerged as a dual metabolic receptor for cardioprotective high-molecular-weight adiponectin and atherogenic low-density lipoproteins. We recently showed that mesenchymal stem/stromal cells lacking T-cadherin are predisposed to adipogenesis, underscoring its role in lineage choice. Emerging evidence indicates that *CDH13* expression and function are fine-tuned by non-coding RNAs (ncRNAs). MiR-199b-5p, miR-377-3p, miR-23a/27a/24-2, and the miR-142 family directly bind *CDH13* 3′-UTR or its epigenetic regulators, affecting transcription or accelerating decay. Long non-coding RNAs (lncRNAs), including antisense transcripts CDH13-AS1/AS2, brain-restricted FEDORA, and context-dependent LINC00707 and UPAT, either sponge these miRNAs or recruit DNMT/TET enzymes to the *CDH13* promoter. Circular RNAs (circRNAs), i.e.circCDH13 and circ_0000119, can add a third level of complexity by sequestering miRNA repressors or boosting DNMT1. Collectively, this ncRNA circuitry regulates T-cadherin across cardiovascular, metabolic, oncogenic, and neurodegenerative conditions. This review integrates both experimentally validated data and in silico predictions to map the ncRNA-*CDH13* crosstalk between health and disease, opening new avenues for biomarker discovery and RNA-based therapeutics.

## 1. Introduction

T-cadherin (also named cadherin-13, H-cadherin) is encoded by the *CDH13* gene. The *CDH13* gene is highly conserved across species, underscoring its evolutionary importance. Human T-cadherin was initially mapped to chromosome 16q24 [1], yet its location was later refined to 16q24.2 [2]. In humans, the *CDH13* gene consists of 1,169,627 base pairs and comprises 14 exons. It produces a 3711-base pair transcript that encodes a 713-amino acid pre-proprotein. This pre-protein features a carboxy-terminal glycosylphosphatidylinositol (GPI)-tethered protein comprising a pro-peptide sequence and five extracellular calcium-binding domains (EC1–EC5) [3,4].

T-cadherin belongs to the cadherin superfamily, whose intricate structural diversity underpins an array of various biological functions [5]. “Classical” cadherins are well-known for mediating cell–cell adhesion, typically through homophilic binding between cadherin molecules. The extracellular domains of “classical” cadherins, which are transmembrane glycoproteins, are characterized by the presence of five extracellular Ca^2+^-binding domains, transmembranes, and extracellular parts. Stable cell–cell adhesion in organs and tissues results from interactions between cadherin cytoplasmic domains, catenins, and actin cytoskeleton [6]. Yet, even E-cadherin, the first discovered member of the cadherin superfamily, is now considered far more than a simple molecular “glue”, as it has been linked to various signaling cascades that drive embryonic development, tissue morphogenesis, and homeostasis [6].

T-cadherin is a non-canonical member of the cadherin superfamily. Although T-cadherin possesses a hallmark extracellular part of “classical” cadherins (five Ca^2+^-binding domains), it lacks both transmembrane and cytoplasmic parts, and is tethered to the plasma membrane via a glycosyl-phosphatidylinositol (GPI) anchor. Moreover, T-cadherin lacks the signature His-Ala-Val (HAV) motif that mediates the homophilic binding of “classical” cadherins, underscoring its distinctive structural and functional profile [6]. T-cadherin ensures only weak homophilic binding and provides negative guidance cues in the developing nervous system, as well as in physiological angiogenesis and tumor-driven neovascularization [7]. This GPI anchor endows T-cadherin with rapid lateral mobility within the plasma membrane and transient interactions with diverse signaling partners [5,6,7].

Mounting evidence indicates that T-cadherin participates in multiple intracellular signaling cascades [3]. Consistent with this, T-cadherin is involved in several intracellular signaling pathways that regulate apoptosis, proliferation, differentiation, migration, and tissue regeneration [3]. Essential T-cadherin-dependent signaling pathways have been identified, including Grp78/BiP/integrin-β3 [8] and PI3K/mTOR [9], which trigger pro-survival signaling and promote endothelial cell proliferation and migration. T-cadherin contributes to angiogenesis, modulating endothelial cell polarization and promoting de-adhesion via homophilic T-cadherin-mediated interaction and activation of RhoA-ROCK and Rac signaling [3]. Furthermore, it suppresses endothelial cell migration, capillary sprouting, and capillary-like tube formation in in vitro, ex vivo, and in vivo models. Under oxidative stress conditions, the overexpression of T-cadherin in cultured endothelial cells protects them from apoptosis [10].

Over the past three decades, multiple studies have underscored the relevance of T-cadherin in tumor biology. *CDH13* often restrains cancer cell proliferation, invasion, and overall tumor growth, yet paradoxically supports tumor progression by promoting neovascularization [3].

Many biological functions are mediated through T-cadherin, which serves as a receptor for two metabolically important ligands, adiponectin and LDL, which may compete for receptor binding in both physiological and pathological contexts [7]. The interaction between adiponectin and T-cadherin is critically important for localizing adiponectin within organs and tissues, thereby facilitating adiponectin’s protective roles against vascular atherosclerosis and myocardial ischemia [11,12,13]. Tanaka et al. demonstrated that adiponectin promotes muscle regeneration specifically through binding to T-cadherin [14]. In addition, Obata et al. reported that the adiponectin/T-cadherin system enhances exosome biogenesis and secretion, leading to a decrease in cellular ceramide levels in cultured endothelial cells and in the aorta in vivo [15]. Plasma adiponectin levels can dramatically decrease in metabolic syndrome, while elevated LDL may bind to T-cadherin, correlating with the LDL-mediated atherogenic impact on the cardiovascular system [16]. We recently demonstrated that mesenchymal stem/stromal cells lacking full-length T-cadherin spontaneously undergo adipogenic differentiation and produce adipocytes with conspicuously large lipid droplets, a propensity that persists in adipogenic conditions [17]. In ligand-comparison experiments, we found that the absence of T-cadherin rendered cells more susceptible to adiponectin’s suppressive effects and LDL’s stimulatory effects on adipogenesis, suggesting that T-cadherin may serve as an important metabolic sensor [17].

The atypical molecular structure of T-cadherin raises compelling questions about how T-cadherin transmits signals and orchestrates such a broad range of biological functions, with the underlying mechanisms likely extending far beyond simple protein–protein interactions between T-cadherin and other membrane receptors or signaling molecules. One promising area of investigation involves non-coding RNAs (ncRNAs), which may regulate the expression and activity of both the T-cadherin protein and its mRNA, as well as the functional activity of its targets and interaction networks. Uncovering the mechanisms governing T-cadherin expression and *CDH13* gene activity offers valuable insights into T-cadherin’s involvement in a wide range of physiological and pathological processes.

Thus, the present review integrates growing evidence that microRNAs (miRNAs), long non-coding RNAs (lncRNAs), and circular RNAs (circRNAs) form an intricate, multilayered network converging on the atypical member of the cadherin superfamily, *CDH13*. We outline how these ncRNAs govern *CDH13*/T-cadherin, from promoter methylation and mRNA stability to ligand presentation, and illustrate that their dysregulation is manifested through cardiovascular, metabolic, oncogenic, and neurological pathways. By assembling both experimentally validated findings and bioinformatically predicted interactions, we highlight the ncRNA/*CDH13* axis as a nexus and a promising source of new biomarkers and therapeutic targets.

## 2. T-Cadherin/*CDH13* Expression Landscapes in Development, Adult Tissues, and Disease

T-cadherin is most prominently expressed in the nervous and cardiovascular systems [3,7,18]. Its expression pattern changes across embryogenesis and in adults, both in health and disease.

T-cadherin is among the earliest cadherins detected in the developing nervous system (E8.5–9.5 in mouse) and nascent vasculature (≈E11.5) [19]. Being differentially expressed in somite sclerotomes, T-cadherin acts as a negative guidance cue defining pathways for migrating neural crest cells and growing axons of motor neurons, as well as ensuring precise synapse formation. In adults, T-cadherin remains widespread throughout the central nervous system (CNS), being robust in the human adult cerebral cortex, medulla, thalamus, and midbrain, but absent from the spinal cord. T-cadherin is expressed at levels even higher than in the developing brain (cerebral cortex, medulla oblongata, and inferior olivary nucleus), but not in the spinal cord, with its expression being even higher than in the developing brain [20].

During cardiovascular development, T-cadherin/*CDH13* localizes to the forming vascular plexus, coincident with intense vessel sprouting [19]. In adult tissues, T-cadherin is highly enriched in the heart and large arteries (aorta, carotid, iliac, and renal arteries), specifically detected in cardiomyocytes, endothelial cells, vascular smooth muscle cells (VSMCs), and perivascular cells [21]. T-cadherin expression rises further in endothelial cells, VSMCs, and pericytes within atherosclerotic plaques and after arterial injury, particularly during neointima formation [22]. In contrast, T-cadherin is modestly expressed in the lungs, skeletal muscle, and kidneys, and is virtually absent in the liver or pancreas [21].

In the skin, strong T-cadherin expression in the basal layer of keratinocytes is lost upon malignant transformation, paralleling its downregulation observed in many carcinomas (breast, lung, and cutaneous squamous cell carcinomas), pituitary adenomas, B-cell lymphomas, and nasopharyngeal carcinoma [23].

T-cadherin is abundantly present in the basal layer of keratinocytes in normal skin and its expression is lost upon malignant transformation as well as in numerous human cancers such as breast and lung carcinomas, cutaneous squamous cell carcinomas, pituitary adenomas, malignant B cell lymphomas and nasopharyngeal carcinoma, osteosarcoma, ovarian cancer and endometrial cancer, and gallbladder cancer [3,18]. Conversely, certain mesenchymal and neural malignancies (osteosarcoma, NF1-deficient astrocytoma, and subsets of hepatocellular carcinoma) retain or even upregulate T-cadherin [3].

Compared to the nervous and cardiovascular systems, adipose tissue displays lower overall T-cadherin levels, yet it is clearly present in the stromal-vascular fraction, notably in mesenchymal stem/stromal cells and adipocyte progenitors, suggesting its role in cellular self-renewal and tissue homeostasis [7]. Table 1 summarizes T-cadherin’s key structural features, its tissue-specific expression, and functional roles in development and adulthood, both in health and disease.

## 3. Non-Coding RNAs: A General Overview

Non-coding RNAs (ncRNAs) are a novel class of RNA molecules that do not encode proteins. The non-coding transcripts are now recognized for their critical roles in epigenetic regulation, including gene silencing, DNA methylation, chromatin remodeling, and gene regulation. NcRNAs regulate a wide range of biological processes, making them promising therapeutic targets as well as biomarkers [42,43]. Dysregulated expression of ncRNAs is associated with various pathological conditions, including cancer.

Regulatory non-coding RNAs are generally classified into two main categories: small non-coding RNAs (sncRNAs) (less than 200 nucleotides) and long non-coding RNAs (lncRNAs) (exceeding 200 nucleotides). In the present review, we narrow our focus down to three major ncRNA categories: miRNAs, lncRNAs, and circRNAs, with a particular focus laid on T-cadherin/(*CDH13*) expression and function [43,44].

NcRNAs can originate from both intronic and exonic regions of protein-coding and non-coding genes, as well as from intergenic areas, transposable elements, and gene promoter regions [44,45,46]. Some lncRNAs are transcribed from their dedicated promoters, while others are synthesized from introns of the neighboring protein-coding loci [47,48]. CircRNAs are derived from back-splicing of lncRNAs of pre-mRNA [49]. In most cases, sncRNAs are excised from longer RNA transcripts by RNAase III-family nucleases, whose double-stranded RNA-binding and catalytic domains guide and cleave the substrate [50]. However, approximately one-third of intronic miRNAs possess independent promoters and can be transcribed separately from their host genes [51].

MiRNAs are short, single-stranded RNA molecules that regulate gene expression by binding to target mRNAs at their 3′ untranslated regions (3′-UTRs) to suppress stable translation [52]. MiRNAs maintain tissue homeostasis and support the differentiated state of cells in various organs and tissues [53]. Additionally, they participate in intercellular communication as components of exosomes, microvesicles, apoptotic bodies, lipoproteins, and ribonucleoproteins [54].

The biological functions of lncRNAs include the regulation of the chromatin architecture, transcription, splicing (particularly by antisense lncRNAs), translation, protein localization, and various RNA processing events, as well as RNA editing, localization, and stability [55]. Numerous lncRNAs orchestrate cell differentiation and development in both animals and plants. They are also involved in regulating critical biological processes, including the p53-mediated DNA damage response, cytokine expression, endotoxic shock, inflammation, neuropathic pain, and cholesterol biosynthesis, to name just a few [45].

Circular RNAs (circRNAs) are long, non-coding endogenous RNA molecules with a covalently closed continuous loop structure that lacks both a 5′ cap and a 3′ poly(A) tail [56]. CircRNAs function as molecular sponges for miRNAs, sequestering them and thereby preventing the degradation of their target messenger RNAs. Emerging evidence also suggests that circRNAs can exert both positive and negative regulatory effects on the transcription of their parental genes [57,58,59]. CircRNAs have been shown to regulate gene expression by interacting with the RNA polymerase II (Pol II) complex in the nucleus or by directly interacting with RNA-binding proteins in the cytoplasm, forming RNA–protein complexes that modulate mRNA transcription. Additionally, circRNAs can suppress gene expression by competing with the pre-mRNA splicing machinery, functioning as “mRNA traps” that sequester translation initiation sites or compromise the integrity of mature linear RNAs, ultimately generating fragmented, non-translatable transcripts or inactive protein products. These mechanisms collectively contribute to the reduced expression of target proteins [60].

Recent studies highlight the growing interest in non-coding RNAs (ncRNAs) encoded within intronic or exonic regions of genes, as these molecules may regulate cellular processes through mechanisms independent of the proteins encoded by the same loci [61]. Despite the well-characterized functions of the T-cadherin protein in health and disease, the mechanisms underlying its regulation and the functional roles of the gene locus remain relatively unexplored. The *CDH13* gene also contains non-coding RNAs within its structure, which will be discussed in detail in the following sections.

## 4. Non-Coding RNAs Expressed from the *CDH13* Gene

The miRBase reference database includes numerous human pre-miRNAs; approximately half are derived from intergenic non-coding pri-miRNA transcripts, while the remaining are excised from introns of protein-coding transcripts [62]. A small fraction (about 6%) of human mature miRNAs, annotated in miRbase, originates from multiple pre-miRNAs encoded at different genomic loci. The precise location of promoters has not yet been mapped for most miRNA genes. Some miRNAs reside within the introns of protein-coding genes and therefore share the host gene’s promoter. However, it has been shown that miRNA genes frequently have multiple transcription start sites [63], and the promoters of intronic miRNAs may sometimes differ from those of their host genes [64].

The *CDH13* gene contains transcripts of several miRNAs. One of them, miR-3182, is located within an intron of the *CDH13* gene (chr16:83,508,346-83,508,408) [65]. This miRNA functionally interacts with its targets in a tissue-specific manner, including targets that are also associated with the *CDH13* gene itself. These interactions were examined using the network-wide association study (NetWAS), which evaluates the functional association between gene pairs based on tissue-specific posterior probabilities. This analysis identified 18 targets of intronic miR-3182, which interact with the *CDH13* gene in the cardiac muscle network, and received high NetWAS scores. Among them, in cardiac muscle and endothelial cells, are genes such as *SUB1*, *CCND2*, *ARHGEF37*, *MXRA7*, *DEFB107A*, *DAB2IP*, and *MAGEB10* expressed in cardiac muscle and endothelial cells [66]. While this bioinformatic analysis provides new insight into the regulatory network of the cardiovascular-associated pathways, it still requires further experimental verification.

Gene set enrichment analysis (GSEA) further revealed that the targets of miR-3182 are enriched in biological processes related to modulating the frequency, rate, or strength of cardiomyocyte contraction. These associations point to a broad range of cardiovascular conditions, including cardiac hypertrophy, heart failure, angiogenesis, and endothelial dysfunction, where RAS (rat sarcoma family of small GTP-binding proteins) signaling is central to pathogenesis [67,68].

MiR-3182 is not the only miRNA expressed from the *CDH13* gene. A novel serum miRNA in sepsis patients with varying clinical outcomes was discovered and characterized to be the previously unannotated miR-8058. This miRNA was identified through bioinformatic analysis and subsequently validated by RT-PCR. Although the functions and targets of miR-8058 remain unknown, it was suggested that several miRNAs identified in the study, including miR-8058, may contribute to the pathogenesis of sepsis and hold promise as prognostic biomarkers for diagnostic applications [69,70]. The genomic location of miR-8058 was determined using bioinformatic mapping [71].

In addition to miRNAs, the *CDH13* gene also gives rise to lncRNAs. In search of molecular mechanisms underlying sex-related differences in the frequency of depression, Issler et al. examined the lncRNA expression patterns in several limbic brain regions. They highlighted one human-specific lncRNA, RP11-298D21.1, which was named FEDORA, and which was differentially expressed across females and males [72]. FEDORA is an antisense lncRNA transcribed from the *CDH13* locus. FEDORA was reported to modulate synaptic plasticity by promoting an increased myelin thickness in oligodendrocytes. While its expression may also be altered in neurons, the mechanisms underlying this regulation remain unclear.

Gene ontology analysis revealed the opposing regulatory patterns in Neuro-FEDORA and Oligo-FEDORA transcriptional profiles, highlighting divergent patterns of expression: high in oligodendrocytes and low in neurons. In human studies, FEDORA was significantly upregulated in the cortical brain regions of women, but not men, diagnosed with major depressive disorder, as compared to healthy controls in two independent cohorts. In a mouse model, FEDORA was shown to induce depression-like behavioral abnormalities, yet exclusively in female mice, associated with cell-type-specific changes in synaptic function, myelination, and gene expression. The authors proposed that FEDORA may exert these effects, in part, through its host gene *Cdh13*, which contributes to divergent gene regulatory patterns across different neural cell types [72].

Another lncRNA, named RP11-543N12.1 and also known as CDH13-AS1 (*CDH13 antisense RNA 1*), was shown to be transcribed from the *CDH13* gene [73]. LncRNAs can compete with endogenous miRNAs for common miRNA-binding sites on target mRNAs, thereby relieving miRNA-mediated repression of gene expression [74]. This competitive mechanism was demonstrated for CDH13-AS1 and miR-324-3p, further supported by predictions from the miRDB4.0 and PITA databases, and followed by validation through dual-luciferase reporter assays. The analysis showed that miR-324-3p is a direct target of CDH13-AS1, and its expression was significantly upregulated when CDH13-AS1 was directly bound to the 3′ untranslated region (3′-UTR) of miR-324-3p. Moreover, the authors demonstrated that CDH13-AS1 targeted miR-324-3p to suppress proliferation and promote apoptosis in an Alzheimer’s disease cell model in vitro, suggesting that CDH13-AS1 and miR-324-3p may be potential biomarkers and therapeutic targets for Alzheimer’s disease. A similar effect was observed in SH-SY5Y cells: upregulated CDH13-AS1 and miR-324-3p led to downregulated proliferation and induced apoptosis. Conversely, silencing CDH13-AS1 and miR-324-3p increased proliferation and suppressed apoptosis in these cells [73].

Towards that end, miR-324-3p is considered a multifunctional miRNA involved in the proliferation and apoptosis of nasopharyngeal carcinoma cells, and its low expression was associated with poor overall survival and recurrence-free survival of patients. It was demonstrated that miR-324-3p can modulate the growth and survival of nasopharyngeal carcinoma cells by upregulating SMAD7 expression [75].

Another illuminating example involves CDH13-AS2, an lncRNA transcribed from the *CDH13* gene, which targets its own mRNA. CDH13-AS2 was predicted to bind the 3′-UTR of *CDH13* using LncRRIsearch and RNAup servers. This binding interaction was further validated by dCas13-mediated RNA immunoprecipitation. Weighted correlation network analysis (WGCNA) of transcriptomic data from patients with CAD revealed a positive correlation between the expression of the *CDH13* gene and its antisense lncRNA *CDH13-AS2* in arterial tissues. Functional validation using CRISPR/Cas9-mediated knockout of either *CDH13* or *CDH13-AS2* in human umbilical vein endothelial cells (HUVECs) resulted in synergistically pro-atherogenic effects, including impaired proliferation and migration, increased apoptosis, and enhanced monocyte adhesion. These findings were consistent with the observed downregulation of *CDH13* in arterial tissues from patients with atherosclerosis. Accordingly, these in vitro data show that *Cdh13* and *Apoe* double-knockout mice *(Cdh13-/-/Apoe-/-)* exhibit increased aortic lesions under a high-fat diet compared to *Apoe-/-* controls. Target prediction tools (TargetScan, miRWalk, and scanMiRApp) identified several endothelial-enriched miRNAs, including miR-let-7, miR-30, and miR-125, with predicted binding sites in the 3′UTR of *CDH13*, some of which overlap with disease-associated variants. A dual luciferase RNAi assay confirmed the direct binding of miR-let-7, while CRISPR/dCas9-mediated activation of *CDH13-AS2* reduced this binding and increased *CDH13* expression [76].

Interestingly, the *CDH13* gene also harbors a circular RNA known as CircCDH13 (*hsa_circ_0040646*) [77], which is formed through back-splicing of exons 9 and 10 of the *CDH13* gene [78]. CircCDH13 was found to be significantly upregulated in chondrocytes of osteoarthritic cartilage and contributed to the disease progression by promoting chondrocyte apoptosis, enhancing extracellular matrix (ECM) catabolism, and suppressing ECM anabolism. In vitro and in vivo experiments, employing gain-of-function and loss-of-function approaches, including RNA pulldown, luciferase assays, and adeno-associated virus-mediated delivery of CircCDH13 in a mouse model of destabilization in medial meniscus-induced osteoarthritis, defined the important role of CircCDH13 in osteoarthritis. Mechanistically, CircCDH13 promotes osteoarthritis progression by acting as a molecular sponge for miR-296-3p, thereby modulating the miR-296-3p-PTEN signaling axis. Specifically, CircCDH13 overexpression enhanced apoptosis through the upregulation of PTEN signaling, which is mediated by miR-296-3p sequestration.

In addition to chondrocytes from osteoarthritic tissue, the elevated expression of CircCDH13 was detected in chondrocytes treated with IL-1β and TNF-α, ultimately linking CircCDH13 expression and function to inflammation [78]. CircCDH13 knockdown resulted in the suppression of matrix metalloproteinase 13 (MMP13) and ADAMTS5 (a disintegrin and metalloproteinase with thrombospondin motifs 5), along with the induction of collagen type II alpha 1 (COL2A1) and aggrecan. Overall, silencing CircCDH13 alleviated osteoarthritis symptoms and restored cartilage homeostasis. These findings highlight CircCDH13 as a critical regulator of osteoarthritis pathogenesis and a promising therapeutic target [77].

**Figure 1:**
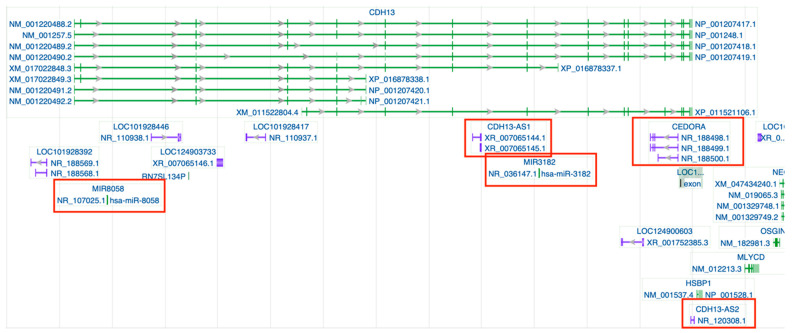
Genomic regions and transcripts of *CDH13* indexed in the NCBI Gene ID database https://www.ncbi.nlm.nih.gov/gene/1012#top (accessed on 26 May 2025). Gray arrows within introns mark splice direction. Gene transcripts are shown in green, whereas non-coding RNA loci appear in purple; those highlighted in red are the specific ncRNAs discussed in this review. A complete graphical key is available in the NCBI legend (https://www.ncbi.nlm.nih.gov/tools/sviewer/legends/) (accessed on 26 May 2025).

Non-coding RNAs transcribed from the *CDH13* gene (Figure 1) may contribute to its own regulation and affect the related signaling pathways. However, *CDH13* expression is governed not only by internal regulatory elements but also by a complex interplay of external factors. Interactions between *CDH13* and a whole range of ncRNAs (miRNAs, lncRNAs, and circRNAs), originating from both the *CDH13* locus and other genomic regions, form an intricate regulatory network capable of modulating *CDH13* activity in both enhancing and suppressive directions. Acting in concert, these ncRNAs orchestrate context-dependent modulation of *CDH13*/T-cadherin across cardiovascular, metabolic, neurological, and inflammatory settings.

In the following sections, we explore the non-coding RNAs that directly regulate *CDH13* gene expression, elucidate their mechanisms of action, and unveil the biological implications of these regulatory interactions.

## 5. Regulation of *CDH13*/T-Cadherin Expression via Non-Coding RNAs

T-cadherin expression is orchestrated by multiple factors ranging from transcriptional and post-transcriptional regulation to epigenetic control. Earlier studies demonstrated the elaborate regulation of T-cadherin by growth factors and hormones. Specifically, estradiol, progesterone, EGF, and dexamethasone added to the culture medium of human osteosarcoma cells significantly increased T-cadherin levels, as was shown by Western blotting and qRT-PCR assays [79]. In contrast, in our lab, we demonstrated that EGF, FGF-2, PDGF, and IGF-1 downregulated T-cadherin expression in smooth muscle cells in vitro, while 8-Br-cGMP, forskolin, and TGFβ increased T-cadherin [80,81].

Epigenetic control emerges to play an important role in *CDH13* regulation: hypermethylation of the *CDH13* promoter correlated with *CDH13* silencing and oncogenic transformation across many tumor types [18,82,83,84]. Direct regulation of *CDH13* expression by transcription factors was reported previously for melanoma cells. Bioinformatic analysis with Genomatix revealed consensus binding sequences for BRN2 (a member of the POU family of transcription factors) in the proximal promoter region of *CDH13*. BRN2 is present in normal melanocytes, where it coordinates complex transcriptional events required for normal melanocytic development and homeostasis. Yet, BRN2 expression was elevated in melanoma tissue samples and melanoma cell lines, correlating with the enhanced invasive and metastatic behavior. Ectopic BRN2 expression in BRN2-deficient/T-cadherin-positive melanoma cells led to suppression of *CDH13* promoter activity, whereas BRN2 knockdown in BRN2-positive/T-cadherin-negative cells resulted in re-expression of *CDH13* mRNA and protein [85].

Recent studies revealed that the expression of *CDH13* can be regulated at a post-translational level, specifically by miRNAs. A prominent example is miR-199b-5p, an intragenic miRNA encoded in the *dynamin 1* (*DNM1*) locus on chromosome 9. The mature 5p strand encoded by hsa-miR-199b is highly conserved across multiple species, and bioinformatic analyses predicted that its transcription is independent of its host *DNM1* gene [86]. In gain-of-function assays, transfecting HL-1 cardiomyocytes with a miR-199b mimic markedly suppressed *CDH13* mRNA levels [87].

Suppression of miR-199b was reported in several types of cancer and predicted poor prognosis in hepatocarcinoma, where it directly targets HIF-1α, in breast cancer, where it targets HER2, and in prostate cancer [88,89,90]. The downregulation of miR-199b is strongly linked to imatinib resistance in chronic myeloid leukemia (CML) patients [91], while the loss of miR-199b expression contributes to the acquired chemoresistance in ovarian cancer [92]. Conversely, overexpression of miR-199b-5p in medulloblastoma stem cells depletes CD133^+^ tumor-initiating cells by repressing Notch signaling [93].

Clinically, low miR-199b correlates with myeloproliferation and poor overall survival in acute myeloid leukemia (AML), and its expression can be restored by histone deacetylases (HDAC) inhibitors, promoting cancer cell apoptosis [86]. Although none of the cited studies directly explored a connection between hsa-miR-199b and *CDH13* expression, T-cadherin itself is widely viewed as a tumor suppressor in numerous malignancies (Table 1) [3,18,26]. Conversely, in other cancers, T-cadherin upregulated expression has been associated with elevated tumor aggressiveness, pronounced tumor vascularization, and an increased propensity for metastasis [7,94]. Clarifying whether miR-199b regulates *CDH13* expression during oncogenesis and whether manipulating this miRNA could yield therapeutic benefit represents an intriguing direction for future cancer research.

The pertinent literature indicates that the adiponectin–T-cadherin axis is prominent for adiponectin-mediated effects in skeletal muscles and the cardiovascular system. Adiponectin, a unique adipokine primarily produced by white adipocytes, exerts numerous protective effects: it improves insulin sensitivity, reduces inflammation and cell proliferation, and suppresses both atherogenesis and tumorigenesis [95,96]. T-cadherin operates as a specific receptor for high molecular weight (HMW) adiponectin, the most metabolically active form of adiponectin [11]. Experimental studies demonstrated that T-cadherin sequesters circulating adiponectin to the cardiac and skeletal muscle tissues. T-cadherin expression is a prerequisite for adiponectin’s protective effects, as demonstrated in mouse models of cardiac hypertrophy and ischemia-reperfusion injury [12,24,25]. In T-cadherin-deficient mice, adiponectin was dramatically elevated in the bloodstream, but failed to associate with cardiomyocytes, and these animals sustained infarct sizes after ischemia-reperfusion comparable to those in adiponectin-knockout mice. Adenoviral delivery of adiponectin rescued the cardiac phenotype in adiponectin-knockout mice, yet with no effect in T-cadherin-knockout mice, underscoring the essential role of T-cadherin in adiponectin-mediated cardioprotection [12,24]. A similar scenario has been reported for skeletal muscles [24].

Towards this end, decreased T-cadherin levels were demonstrated in human myocardium in non-ischemic dilated cardiomyopathy (NI-DCM) and chronic heart failure (HF) [97]. While systemic levels of adiponectin were high in these patients, adiponectin failed to exert protective effects, most probably due to the diminished T-cadherin content, which is an additional indicator of HF severity. Beyond sarcomeric proteins, in silico analyses indicated that the adiponectin axis, comprising adiponectin and its receptors, is a putative target for several miRNAs, including miR-199b-5p. Transfecting HL-1 cells (muscle cell line) with a synthetic miR-199b-5p mimic further confirmed this interaction, producing a noticeable reduction in T-cadherin expression [87].

Several independent research groups have shown that miR-377-3p accelerates cell-cycle progression, promoting proliferation, whereas suppressing miR-377-3p results in cell-cycle arrest and apoptosis. For example, in human hepatocellular carcinoma cell lines (Hep3B, HepG2, LM3, Li-7, and HuH-7), miR-377-3p was shown to suppress the transcription factor EGR1, thereby attenuating p53 activation [98]. Interestingly, *CDH13* was identified as a direct target of miR-377-3p in both colorectal cancer and an Alzheimer’s disease model, resulting in its downregulated expression [99,100].

Of note, adiponectin has attracted considerable attention in oncology, as low circulating levels of this adipokine correlate with a high incidence and poor prognosis of several types of cancer. This association is particularly strong for digestive tract malignancies, such as esophageal adenocarcinoma, gastric, liver, and colorectal cancers [101,102,103,104,105,106]. Studies in colorectal cancer cell lines reinforce this link: in HCT116, HT29, and LoVo, adiponectin suppressed proliferation, migration, and clonogenicity [103,104]. It was suggested that adiponectin may inhibit proliferation, migration, and clonogenicity by modulating cell metabolism as well as cell cycle progression [105].

With respect to miR-377-3p, bioinformatic screening performed by Di Palo et al. predicted that this miRNA targets the 3′-UTR of T-cadherin mRNA in colorectal adenocarcinoma (HT-29 and Caco-2) cell lines, downregulating its expression [99]. A dual-luciferase reporter assay confirmed the direct binding, and gain- or loss-of-function experiments showed that a miR-377 mimic markedly reduced T-cadherin protein level, whereas a miR-377 inhibitor restored its expression. An analysis of TCGA data via the ENCORI platform further revealed an inverse correlation between miR-377 and *CDH13* expression in colorectal cancer samples. Functionally, miR-377-3p was demonstrated to accelerate G1-S transition, enhance migration and invasion, and drive epithelial–mesenchymal transition in colorectal cancer through GSK-3β upregulation and NF-κB activation [107]. Together, these data further underscore a miR-377/T-cadherin/adiponectin axis that may contribute to colorectal carcinogenesis and offer a potential avenue for biomarker discovery and therapeutic intervention aimed at restoring T-cadherin expression. Reinforcing this link, miR-377 has recently been shown to aggravate adipose-tissue inflammation and insulin resistance, in which the adiponectin–T-cadherin pathway is similarly dysregulated [108].

With regard to tumors originating from the CNS, T-cadherin was shown to function as a negative regulator of cell proliferation. While T-cadherin expression was elevated during physiological growth arrest of normal astrocytes in vitro, in several cell lines, T-cadherin re-expression decreased cell growth, while enhancing substrate attachment, homophilic adhesion, and reducing motility. T-cadherin re-expression resulted in G2-phase arrest in glioma cells, which coincided with the upregulation of the cyclin-dependent kinase inhibitor p21, but was independent of p53 status [109]. Moreover, TGW and NH-12 neuroblastoma cell lines transfected with a T-cadherin cDNA-expressing vector lost their mitogenic potential and proliferative response to epidermal growth factor (EGF) [20].

Interestingly, miR-377-3p can override T-cadherin-mediated regulation. In an in vitro Alzheimer’s disease model, Liu et al. showed that miR-377 serves as a potent negative regulator of T-cadherin. β-Amyloid-treated SH-SY5Y neuroblastoma cells displayed a robust rise in *CDH13* mRNA and protein levels, accompanied by a pronounced drop in endogenous miR-377. A markedly reduced *CDH13* expression was detected upon transfection with miR-377 mimics, whereas anti-miRNA-377 produced the opposite effect, correlating with T-cadherin protein and mRNA expression levels. Functionally, the elevated miR-377 enhanced cell proliferation and reduced apoptosis in the β-amyloid-treated neurons. These findings confirm a negative regulatory relationship between miR-377 and T-cadherin in β-amyloid-challenged neurons [100].

Non-coding RNAs typically act within complex regulatory networks, which are interconnected molecular components that interact with one another and with their target genes to orchestrate gene expression. miRNAs that regulate *CDH13* expression and function form part and parcel of this dynamic system. For example, H19, an lncRNA, is among the most abundant and evolutionarily conserved transcripts in mammalian development, with expression in both embryonic and extraembryonic cell lineages. The oncogenic role of H19 is now well recognized: aberrant H19 expression correlates with tumor growth, proliferation, invasion, metastasis, treatment resistance, and poor patient outcomes in several cancers [110]. For example, high-grade gliomas display elevated H19 levels, whereas siRNA-mediated knockdown of H19 suppresses glioma-cell invasion. miR-675 is embedded within the H19 first exon [111]. High H19 expression demonstrated a positive association with miR-675, and silencing H19 concomitantly inhibited miR-675 expression. Bioinformatic screening with miRanda and PicTar revealed a perfect seed-match between miR-675 and the 3′-UTR of *CDH13*, while luciferase assay using *CDH13* 3′-UTR constructs confirmed that miR-675 directly targets *CDH13*. Re-expression of miR-675 reversed the anti-invasive effect of H19 knockdown, demonstrating that miR-675 modulates *CDH13* by binding its 3′-UTR [112].

Moreover, H19 functions as a competing endogenous RNA that sequesters miR-138 and miR-200a and antagonizes their functions. Inhibition of H19 has been shown to repress MMP9, vimentin, E-cadherin, and *CDH13* expression in retinoblastoma [113], colorectal, and gastric cancers [114], thereby contributing to epithelial to mesenchymal transition progression [76]. H19 is not the only lncRNA that modulates *CDH13* expression. Using LncRRIsearch and RNAup for bioinformatic analysis, CDH13-AS2, a lncRNA transcribed from the *CDH13* locus, was identified as a candidate binder of the *CDH13* 3′-UTR. dCas13-based RNA immunoprecipitation subsequently verified this interaction, showing that CDH13-AS2 negatively regulates *CDH13*. Weighted correlation network analysis of human CAD datasets (STARNET and GTEx) further revealed a positive correlation between *CDH13* and CDH13-AS2 expression in arterial tissue. CRISPR/Cas9 knockout of *CDH13* or CDH13-AS2 in HUVECs produced concordant atherogenic phenotypes, suppressed proliferation and migration, coupled with an increased apoptosis and monocyte adhesion. In silico screens (TargetScan, miRWalk, and scanMiRApp) mapped several endothelial miRNAs to the CDH13 3′-UTR. Several disease-associated variants were mapped to the predicted binding sites of a few miRNAs, including mir-let7. The dual luciferase-based RNA interference assay confirmed the binding of mir-let7 on 3′UTR of *CDH13*, while dCas9-driven activation of CDH13-AS2 weakened the binding of mir-let7 and enhanced *CDH13* expression. Collectively, these data position *CDH13* and CDH13-AS2 as co-regulated, while their inhibition may contribute to CAD. Functionally, CDH13-AS2 stabilizes *CDH13* mRNA, partly by preventing mir-let7-mediated *CDH13* mRNA decay, highlighting the therapeutic potential of targeting the CDH13-AS2/let-7/CDH13 axis [76].

Therefore, the above-mentioned data indicate that *CDH13*/T-cadherin is controlled by an intricate ncRNA circuitry, including miRNAs, antisense and intergenic lncRNAs, and circRNAs, that operates alongside classic transcription-factor and promoter-methylation cues to fine-tune its expression. Disruption of this ncRNA–CDH13 axis spreads through cardiovascular, metabolic, oncogenic, and neurodegenerative pathways, underscoring its value as both a mechanistic understanding and a promising therapeutic target.

## 6. Non-Coding RNAs That Indirectly Regulate *CDH13*

Non-coding RNAs can modulate *CDH13* expression not only through direct mRNA binding but also through engaging the epigenetic machinery that controls *CDH13* transcription. A key pathway involves the opposing activities of two enzymes: DNMT3B and TET1. DNMT3B, a DNA methyltransferase that is frequently overexpressed in hepatocellular carcinoma, breast, prostate, colorectal, and lung cancers and predicts poor clinical outcome. TET1, a DNA demethylase that is often downregulated in the same cancer types, including breast, liver, lung, pancreatic, and prostate malignancies [115,116].

**Figure 2:**
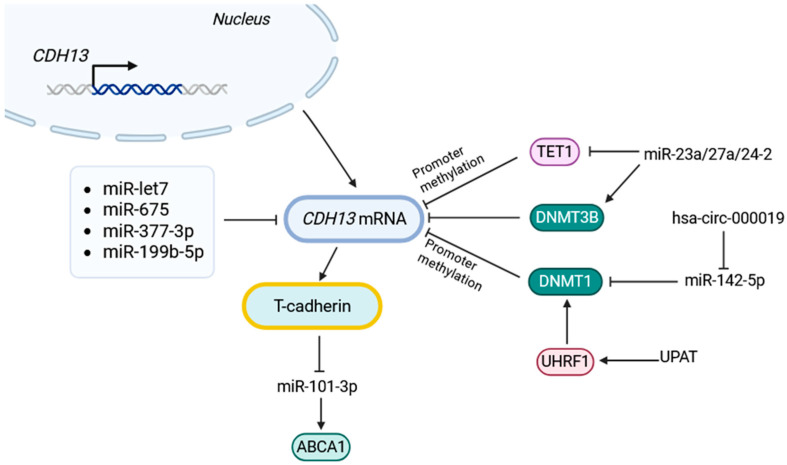
Several miRNAs directly bind the *CDH13* 3′-UTR and repress T-cadherin synthesis, notably miR-377-3p, miR-675, miR-199b-5p, and the let-7 family [76,87,99]. The miR-23a/27a/24-2 cluster silences *CDH13* through an indirect route: it suppresses the demethylase TET1, while elevating the methyl-transferase DNMT3B, shifting the balance toward promoter hypermethylation and transcriptional repression [119]. Beyond miRNAs, circRNA circ_0000119 and lncRNA UPAT also converge on DNMT1/3-dependent methylation, reinforcing *CDH13* repression [121,122]. Conversely, T-cadherin can influence the non-coding landscape itself: for example, it can lower miR-101-3p, thereby lifting inhibition of the cholesterol transporter ABCA1 [124]. Together, these layers of direct binding and epigenetic crosstalk intertwine *CDH13* into a dynamic ncRNA network that fine-tunes its expression in embryogenesis and disease.

An increase in DNMT3B activity and/or a decrease in TET1 activity leads to promoter hypermethylation, consequently suppressing *CDH13* (T-cadherin) expression (Figure 2) [117,118,119].

Certain miRNAs can modulate DNMT3B and TET1 activity. Simultaneous overexpression of the miR-23a/27a/24-2 cluster is an independent predictor of postoperative recurrence and poor survival at the early stage of non-small cell lung cancer (NSCLC). Mechanistically, each miRNA in the cluster targets the 3′-UTR of TET1 and FOXO3, the DNMT3B repressor: direct binding suppresses TET1, while relieving the FOXO3-mediated restraint of DNMT3B shifts the TET1/DNMT3B balance toward gene promoter hypermethylation. Immunohistochemical and Western blot analyses in xenografts confirmed the downstream effects, including the loss of p16 and T-cadherin (*CDH13)* expression along with DNMT3B upregulation. Accordingly, in vitro and in vivo studies have shown that the enforced miRNAs cluster expression heightens Wnt/β-catenin signaling and enhances stemness, tumorigenicity, and metastasis, whereas inhibition of all three miRNAs reverses those traits. Thus, the miR-23a/27a/24-2 cluster epigenetically silences multiple tumor suppressor genes, including *CDH13*, establishing a feed-forward circuit accelerating NSCLC progression and offering a rationale for multi-miRNA blockade as an adjuvant strategy to prevent early relapse [119].

A similar DNMT1-related mechanism, triggering hypermethylation of the *CDH13* promoter and suppressing T-cadherin protein expression, was demonstrated for miR-142-3p [120]. This miRNA drives a positive-feedback circuit, in which it silences DNMT1 repressors and stabilizes DNMT1 mRNA, thereby shifting the DNMT1/TET balance towards net DNA methylation. Elevated DNMT1 then hypermethylates CpG-rich promoters of the key tumor suppressors, including *CDH13*, hampering their transcription and disrupting cell–cell adhesion across multiple malignancies (lung, gastric, and breast). Forced miR-142-3p expression drives proliferation, migration, and chemoresistance in lung cancer and hepatoma lines, whereas miR-142-3p knockdown or pharmacological DNMT1 inhibition restores *CDH13* expression and sensitivity to apoptosis induction. Functionally, the overexpression of miR-142-3p results in invasive growth, whereas its inhibition, or DNMT1 knockdown, suppresses metastasis. Thus, the miR-142-3p/DNMT1/CDH13 cascade represents a convergent epigenetic switch that can be exploited in two ways. Therapeutically, by antagonizing miR-142-3p, or pharmacologically, by suppressing DNMT1, could restore *CDH13* expression and inhibit tumor progression [120].

MiRNAs are not the only non-coding RNAs that can regulate *CDH13* through promoter methylation. In ovarian cancer, circ_0000119 and DNMT1 have been reported to be upregulated, whereas miR-142-5p is reported to be depleted. Functional assays show that circ_0000119 overexpression accelerates cell proliferation, migration, invasion, and in vivo tumor growth, whereas its silencing produces the opposite effects. Functionally, circ_0000119 acts as a sponge for miR-142-5p, thereby releasing DNMT1 from miRNA-mediated repression. The consequent rise in DNMT1 hypermethylates the CpG-rich *CDH13* promoter, thus blocking T-cadherin tumor-suppressive activity. miR-142-5p, in turn, can interact with hsa_circ_0000119 and DNMT1 3′-UTR. Silencing of DNMT1 can reverse the inhibition of hsa_circ_0000119 and circ_0000119-mediated repression of miR-142-5p and *CDH13*. Clinically, *CDH13* promoters are more heavily methylated in ovarian tumors than in normal tissue, and a DNA-methyltransferase inhibitor can reactivate *CDH13* expression in cancer cells and its anti-tumor functions [121].

Another non-coding RNA that represses *CDH13* is the long non-coding RNA UPAT (ubiquitin-like with PHD and RING finger domains 1 (UHRF1) protein-associated transcript). UPAT is overexpressed in several malignancies, including NSCLC and colorectal cancer [122,123], where its levels correlate with tumor size and tumor-node metastasis stage. In NSCLC cells, UPAT markedly promotes proliferation and G1-to-S phase transition, counteracting the growth arrest normally enforced by T-cadherin via the cyclin-dependent kinase-inhibitor-1 pathway. UPAT acts via a more intricate mechanism than the circRNAs described above: UPAT upregulates UHRF1 (ubiquitin-like with PHD and RING finger domains 1), preventing its ubiquitin-mediated degradation. The accumulated UHRF1 recruits DNMT1 to replicating DNA, enhances CpG methylation across the *CDH13* promoter, and inhibits T-cadherin expression. Silencing UPAT, therefore, restores T-cadherin, thereby restraining tumor growth [122].

It is noteworthy that T-cadherin/*CDH13* itself is capable of regulating miRNAs. A recent study highlighted a compelling interplay among T-cadherin, miR-101-3p, ATP-binding cassette transporter A1 (ABCA1), and C1q/TNF-related protein 15 (CTRP15) in atherosclerosis progression [124]. CTRP15 (myonectin) is a myokine within the highly conserved CTRP family of adiponectin paralogues that links skeletal-muscle activity to lipid homeostasis in liver and adipose tissue in response to the energy-state changes, thereby outlining a novel myonectin-mediated metabolic circuit [125]. CTRPs engage three receptors (adiponectin receptors (AdipoR1 and AdipoR2), and T-cadherin), yet CTRP15 overexpression in THP-1 macrophages selectively elevated T-cadherin (protein and mRNA) without altering AdipoR1 or AdipoR2. Functionally, CTRP15 promotes the reverse-cholesterol transport and reduces atherosclerotic lesions in ApoE^−^/^−^ mice. Moreover, CTRP15 enhanced cholesterol efflux from macrophages and increased ATP-binding cassette transporter A1 (ABCA1) expression via the T-cadherin/miR-101-3p axis. miR-101-3p itself is a pleiotropic miRNA implicated in atherosclerosis, cardiovascular disease and metabolic disorders [126,127].

Bioinformatic analyses with miRDB and TargetScan revealed a putative miR-101-3p binding site within the ABCA1 3′-UTR. CTRP15 upregulates ABCA1 and cholesterol efflux by downregulating miR-101-3p. Importantly, siRNA-mediated inhibition of T-cadherin abolished the CTRP15-induced decrease in miR-101-3p, demonstrating that T-cadherin is essential for CTRP15-dependent miR-101-3p suppression, ABCA1 induction, enhanced cholesterol efflux, and subsequent attenuation of atherosclerotic progression. This may indicate the existence of as-yet-undiscovered regulatory mechanisms between T-cadherin and miRNAs [124].

Collectively, a diverse set of miRNAs, circRNAs and lncRNAs modulate *CDH13* indirectly by shifting the DNMT vs. TET balance toward (or away from) promoter hypermethylation, thereby silencing or rescuing T-cadherin transcription. Given that this ncRNA epigenetic circuitry governs *CDH13* expression across cardiovascular and metabolic disorders and cancer, targeting these key ncRNA pathways offers a promising strategy to reestablish T-cadherin/*CDH13* protective functions in disease.

## 7. Bioinformatically Predicted Non-Coding RNAs Regulating *CDH13*/T-Cadherin

Bioinformatically predicted interactions between non-coding RNAs (ncRNAs) and *CDH13*/T-cadherin collectively draw the compelling conceptual landscape for future research aimed at elucidating physiological and pathological mechanisms. Yet, due to the low binding specificity of ncRNAs to their targets, various databases ultimately yield thousands of predicted partners, with most of them still awaiting experimental confirmation [128]. Even so, a subset of high-confidence candidates clearly warrants closer scrutiny.

One miRNA of interest is miR-495-3p, which has been implicated in the pathogenesis of gastric cancer [129]. In their study, Wang et al., retrieved miRNAs predicted to target dysregulated cadherins from miRDB, and cross-checked these miRNA–cadherin pairs in miRTarBase, followed by refining the list with TCGA (The Cancer Genome Atlas) miRNA-seq data and retaining only miRNAs differentially expressed between gastric cancer and normal tissue. miR-495-3p emerged from this pipeline as a putative regulator of several cadherins, including *CDH13*, but its own expression is reduced in gastric cancer. miR-495-3p may represent an attractive yet still unverified component of the *CDH13* regulatory network, and direct binding, along with functional assays, are warranted to define its impact on T-cadherin expression.

Another candidate is miR-584-5p, described as a marker of pathological states in coronary artery disease (CAD), which was demonstrated to target *CDH13* [130]. In a Turkish cohort, whole-blood miRNA profiling of angiographically verified CAD patients versus controls revealed several miRNA candidates, where miR-584-5p appeared to be one of the most dysregulated miRNAs. These microarray findings were further validated by qRT-PCR, followed by diagnostic and clinical correlation assays. Ingenuity pathway analysis defined *CDH13* as the sole CAD-related predicted target of miR-584-5p. These data highlight miR-584-5p, previously recognized as a tumor-suppressor miRNA, as a potential CAD biomarker and implicate this miRNA and *CDH13* in the disease pathogenesis.

A group of miRNAs (miR-181d, miR-30a-3p, miR-30c, miR-30d, miR-30e-3p, miR-370, miR-493-5p, and miR-532-5p) has been associated with ovarian carcinoma progression and predicted to target *CDH13*. Their expression was quantified by TaqMan RT-qPCR in formalin-fixed, paraffin-embedded ovarian tumor samples and in six normal human ovarian surface-epithelial (HOSE) cell lines. Subsequent RT-qPCR analyses of ovarian carcinoma tissues revealed that in Her2/neu-negative tumors, *CDH13* was silenced through miRNA-mediated mechanisms, whereas in Her2/neu-positive tumors, these miRNAs were suppressed and *CDH13* was repressed via promoter methylation. Overall, these data indicated the *CDH13* downregulation across ovarian carcinoma subtypes. The above-mentioned miRNA levels also correlated with key clinicopathologic parameters. Although direct binding to *CDH13* calls for experimental confirmation, TargetScan predicted interaction sites for each of these miRNAs [131,132].

Ducoli and colleagues combined RNA-seq, CAGE-seq, and ChIRP-seq to map the lncRNA landscape of human dermal blood (BEC) and lymphatic endothelial cells (LEC) [133]. LET1R, an endothelial-specific lncRNA, was predicted to be involved in regulating cell proliferation and migration. Bioinformatic mapping placed LET1R-binding motifs within promoters, exons, or introns of 1607 protein-coding genes; T-cadherin (*CDH13*) was among the predicted targets and appeared to be repressed by LET1R. Filtering for genes expressed in endothelial and lymphatic cells, and intersecting this list with the 255 transcripts altered after LET1R antisense-oligonucleotide knockdown, yielded 44 overlapping genes (12 upregulated and 32 downregulated). Among the downregulated set was *CDH13*, suggesting that LETR1 may repress T-cadherin in endothelial cells. These bioinformatic data position LETR1 as a novel lncRNA that potentially modulates the *CDH13* axis in the endothelium and contributes to the emerging network of non-coding RNAs that define T-cadherin biology [133].

Another lncRNA of interest is LINC00707. Transforming growth factor-β (TGF-β) signaling stands at the nexus of tumor-suppressive and tumor-promoting pathways: in early lesions, it blocks proliferation, whereas in advanced cancers, it drives epithelial-to-mesenchymal transition (EMT), migration, and metastasis through a Smad-dependent transcriptional program [134,135]. Genome-wide profiling of TGF-β responses revealed LINC00707 as a direct Smad/MAPK target that is rapidly repressed when TGF-β is activated [136]. Mechanistically, TGF-β lowers the occupancy of the transcription factor KLF6 at the LINC00707 promoter, suppressing its transcription. Loss of LINC00707 then releases cytoplasmic Smad proteins, allowing their nuclear accumulation and activation of the mesenchymal gene program. Although being oncogenic in lung adenocarcinoma and hepatocarcinoma, where its overexpression correlates with increased proliferation, invasion, and a poor prognosis, LINC00707 operates as a negative modulator of TGF-β signaling in other settings. Gene ontology analysis yielding 263 differentially expressed genes (99 upregulated and 164 downregulated) highlighted processes classically associated with a mesenchymal switch: extracellular-matrix assembly, cell–cell adhesion, and cell migration (*FN1*, *COL3A1*, *SERPINE2*, and *CDH13*). *CDH13* was one of the transcripts most consistently elevated when LINC00707 was silenced, yet remained low in cells that ectopically overexpressed LINC00707, indicating that lncRNA normally limits T-cadherin expression. Defining the exact mechanism of LINC00707-mediated repression of *CDH13* and determining whether manipulating this lncRNA can promote mesenchymal-to-epithelial transition and restore *CDH13* function remains an intriguing question for future study [136].

A recent bioinformatic analysis applied to transcriptomic profiling during oocyte maturation in spotted scats used RNA-seq and small-RNA-seq to map circRNA–miRNA–mRNA circuits during IGF3-induced ovarian maturation. Gene ontology (GO) enrichment analysis showed that host genes of differentially expressed circRNAs and target genes of differentially expressed miRNAs were enriched for various processes with a high degree of overlap. Among 176 differentially expressed circRNAs and 52 miRNAs, pathway enrichment highlighted cell adhesion, ECM remodeling, and oocyte meiosis programs. *CDH13* (T-cadherin) appeared consistently among the top Kyoto Encyclopedia of Genes and Genomes (KEGG)-enriched categories, specifically within the “cell-adhesion molecules” and “ECM–receptor interaction” pathways. A circRNA–miRNA–mRNA regulatory network reconstruction identified a single circRNA (Lachesis_group5:6245955|6270787) that is co-expressed in the ovary and is predicted to sponge three IGF3-responsive miRNAs (novel_miR_622, novel_miR_980, and novel_miR_64), thereby affecting a cluster of maturation-related genes that includes *CDH13*. These in silico findings imply that sponging these miRNAs could modulate *CDH13* function during oocyte meiosis and extracellular matrix remodeling throughout teleost ovarian development [137].

In silico screens across diverse datasets converge on a certain list of miRNAs, lncRNAs, and circRNAs that are predicted to bind *CDH13* or its promoter in tissue-specific and disease-related contexts. While these candidates outline a rich regulatory network linking T-cadherin to gastric and ovarian cancer, coronary artery disease, and endothelial and developmental pathways, definitive binding assays and functional studies are warranted to move them from bioinformatically predicted interactions to biological facts.

The growing body of evidence underscores the importance of non-coding RNA interactions with the *CDH13* in both normal and pathological contexts. These findings highlight the regulatory potential of ncRNAs in modulating *CDH13* expression and are summarized in Table 2.

## 8. Conclusions

Mounting evidence positions non-coding RNAs (ncRNAs) as pivotal regulators of *CDH13* gene/T-cadherin at all levels of gene/mRNA/protein expression control, from chromatin architecture to mRNA turnover and protein synthesis. ncRNAs can modulate *CDH13* gene/T-cadherin expression and functions through a variety of pathways. MiRNAs, lncRNAs, and circRNAs can bind directly to the *CDH13* transcript, compete for the shared regulatory elements, or reshape promoter methylation, thereby adjusting *CDH13* gene/T-cadherin output with exquisite precision. Intriguingly, the *CDH13* locus itself encodes several of these regulatory molecules, for example, miR-3182, the antisense lncRNAs FEDORA and CDH13-AS1/-AS2, and CircCDH13, each displaying tissue-restricted expression patterns and establishing feedback loops on the same signaling circuits in which *CDH13* gene/T-cadherin participates.

Collectively, this multilayered ncRNA network fine-tunes physiological programs, such as vascular remodeling, metabolic homeostasis, and neuroplasticity, while its dysregulation contributes to the development of cardiovascular disease, tumor progression, inflammation, and neurodegeneration. These insights make these ncRNAs both informative biomarkers and attractive therapeutic entry points in conditions when *CDH13* gene activity is disrupted.

Yet the landscape remains only partially mapped. Further studies are warranted, including (i) delineating ncRNA-induced chromatin remodeling at the *CDH13* promoter, (ii) constructing cell-type resolved maps of ncRNA/*CDH13* interactomes in vivo, and (iii) converting these insights into clinically viable ncRNA delivery or inhibition strategies. Concerted application of high-resolution multiomics, predictive bioinformatics, and precision functional assays will open new vistas for understanding *CDH13* gene/T-cadherin-related mechanisms in health and disease. Integrating these tools will clarify how ncRNA circuits modulate T-cadherin signaling and pave the way for novel therapies aimed at restoring the *CDH13* expression.

## Figures and Tables

**Table 1:** Structural and functional features of T-cadherin.

T-Cadherin Expression/Key Functional Implications	Tissue/System	Embryonic/Adult	Reference
Negative guidance cue in the developing nervous system	Migrating neural crest cells and motor neuron axons	Embryogenesis	[4]
Widespread throughout the CNS (cerebral cortex, medulla, thalamus, hippocampus, and midbrain), in the medulla oblongata, and the nucleus olivaris	Brain and embryonic CNS	Adult and embryogenesis	[20]
Expression in the heart and large arteries (cardiomyocytes, endothelial cells, VSMCs, and perivascular cells)	Cardiovascular system	Adult	[21]
Negative guidance cue in physiological angiogenesis; suppresses endothelial cell migration, capillary sprouting, and capillary-like tube formation	Endothelial cells (in vitro, ex vivo, and in vivo models)	Adult	[19]
Protects endothelial cells from apoptosis under oxidative stress; regulates apoptosis, proliferation, differentiation, migration, and tissue regeneration	Endothelial cells	Adult (stress)	[3,8,9,10]
Receptor for two metabolically important ligands, adiponectin and LDL, competing for T-cadherin binding	Cardiovascular system	Adult and pathological	[7]
T-cadherin sequesters adiponectin to cardiac and skeletal muscles and promotes regeneration through binding to T-cadherin	Skeletal muscle	Adult	[12,14,24,25]
Adiponectin/T-cadherin system enhances exosome biogenesis and secretion, decreasing cellular ceramide levels	Endothelial cells and aorta	Adult	[15]
T-cadherin expression in the basal layer of keratinocytes is lost upon malignant transformation	Skin (keratinocytes)	Adult and tumors	[23]
Liver, pancreas, thyroid gland, adrenals, spleen, lymph nodes, stomach, oesophagus, small intestine, gall bladder, bladder, lungs, and bronchi low T-cadherin	Secretory and hemogenic organs and tissues	Adult	[21]
T-cadherin deficiency leads to spontaneous MSCs adipogenic differentiation and increases their sensitivity to adiponectin-suppressive and LDL-stimulatory effects on adipogenesis	Adipose-derived MSCs	Adult (in vitro)	[17]
T-cadherin expression rises in atherosclerotic plaques and after arterial injury	Vascular wall	Adult (pathology)	[22]
T-cadherin/*CDH13* loss/silencing correlates with tumor progression	Breast, lung, colorectal, ovarian, endometrial, pancreatic, cervical, nasopharyngeal carcinoma, prostate cancer, retinoblastoma, basal cell carcinoma, cutaneous squamous carcinoma, non-small cell lung carcinoma (NSCLC), gall bladder cancer, and melanoma	Cancer	[3,18,26,27,28,29]
T-cadherin/*CDH13* upregulation correlated with tumor progression	Osteosarcoma, NF1-deficient astrocytoma, and subsets of hepatocellular carcinomas	Cancer	[3,18]
3 novel forms of soluble T-cadherin	Serum	Type 2 diabetes	[30]
Age-related bone loss. T-cadherin in plasma inhibits osteoclast differentiation by blocking RANKL signaling	Plasma	Adult (aging)	[31]
SMC phenotypic modulation by T-cadherin via GSK3β inactivation. Autophagy and survival in VSMCs through MEK1/2/Erk1/2	Vascular smooth muscle cells	Adult (in vitro)	[32,33]
*CDH13* SNPs association with neuropsychiatric disorders (schizophrenia, ASD, ADHS, alcoholism, and sexual behavior)	Blood, mice models (in vivo)	Adult	[34,35,36,37,38,39,40,41]

**Table 2:** Non-coding RNAs interacting with *CDH13*.

miRNA	ncRNA Type	Interaction with *CDH13*	Experimentally Confirmed	Reference
miR-377-3p	miRNA	Direct binding to *CDH13* and suppression of its expression	Yes	[99]
Cluster miR-23a/27a/24-2	miRNA	Inhibits *CDH13* via promoter hypermethylation mediated by suppression of TET1 and activation of DNMT3B	Yes	[119]
miR-142-5p	miRNA	Activates *CDH13* expression by inhibiting hypermethylation through suppression of DNMT1	Yes	[120]
miR-101-3p	miRNA	T-cadherin forms a complex with CTRP15 and suppresses miR-101-3p levels, leading to increased *ABCA1* expression and cholesterol efflux	Yes	[127]
miR-675	miRNA	Direct binding to the 3′-UTR of *CDH13*, modulating its expression	Yes	[112]
miR-let7	miRNA	Direct binding to the 3′-UTR of *CDH13*, suppressing expression	Yes	[76]
miR-199b-5p	miRNA	Direct binding and suppression of *CDH13* expression	Yes	[87]
miR-495-3p	miRNA	Predicted bioinformatically to target *CDH13* in gastric adenocarcinoma, but not experimentally confirmed	No	[129]
miR-584-5p	miRNA	Downregulated in cardiac ischemia tissues; predicted to affect *CDH13* via bioinformatics, yet not experimentally confirmed	No	[130]
Group miR-181d, 30a-3p, 30c, 30d, 30e-3p, 370, 493-5p, and 532-5p	miRNA	Predicted to target *CDH13*; expression profiles in ovarian carcinoma suggest suppression via miRNA direct binding, yet not experimentally confirmed	No	[131]
miR-2419-5P	miRNA	Inverse correlation between miRNA levels and *CDH13* in normal ovarian tissue; binding predicted bioinformatically, not experimentally confirmed	No	[138]
H19	lncRNA		Yes	[110,112,113]
UPAT	lncRNA	Upregulates *UHRF1*, leading to epigenetic suppression of *CDH13* transcription.	Yes	[122]
LET1R	lncRNA	Represses *CDH13* in lymphatic endothelial cells	Yes	[133]
LINC00707	lncRNA	Activates *CDH13* expression in keratinocytes following stable suppression of *LINC00707.*	Yes	[136]
CDH13-AS2	lncRNA	Competes with miR-let-7 for binding to the *CDH13* mRNA, increasing *CDH13* expression	Yes	[76]
